# Voltage faults diagnosis for lithium-ion batteries in electric vehicles using optimized graphical neural network

**DOI:** 10.1038/s41598-025-13188-9

**Published:** 2025-07-27

**Authors:** Jian Ouyang, ZiHao Lin, Liyazhou Hu, Xiaofen Fang

**Affiliations:** 1 Industrial Training Center, Guangdong Polytechnic Normal University, Guangzhou, 510665 Guangdong China; 2 School of Automation, Guangdong Polytechnic Normal University, Guangzhou, 510665 Guangdong China; 3 School of Electronics and Communication Engineering, Shenzhen Polytechnic University, Shenzhen, 518055 China; 4 School of Computer Science and Engineering, Macau University of Science and Technology, Macau, 999078 China

**Keywords:** Lithium-ion batteries, Diagnosis of voltage faults, Optimized graphical neural network, Electric vehicles, Batteries, Computational science

## Abstract

Diagnosing voltage faults of lithium-ion batteries is a critical function in the battery management system. Accurate diagnosis of voltage faults is crucial for ensuring the safety and reliability of energy storage applications and electric vehicles (EVs). This article proposes an optimized Graphical Neural Network (GNN) model. Specifically, the optimized GNN model extracts the relationships between various batteries by learning the topology of the batteries. The proposed method combines the physical coupling between batteries and the entanglement of measurement results with the strong nonlinear processing capability of neural networks to improve the effectiveness of fault localization. Experimental results on three publicly available datasets show that the proposed method outperforms baseline methods such as GraphConv, GCNConv, ChebConv, SGConv, CNN, DBN, LSTM and CNN-LSTM in terms of Accuracy, Precision, Recall, and F1-score, which verifies the effectiveness and accuracy of the proposed method in fault localization of voltage data. Compared to the highest-performing baseline method, the proposed method achieves a maximum improvement of 4.31% and 3.68% in the accuracy of abrupt fault and gradual fault localization respectively. This indicates that the proposed optimized GNN method for diagnosing voltage faults has satisfactory accuracy and stability, which is of remarkable significance for the development of EVs.

**Supplementary Information:**

The online version contains supplementary material available at 10.1038/s41598-025-13188-9.

## Introduction

In order to reduce carbon emissions and decrease the consumption of fossil fuels, countries around the world have taken measures to lower energy consumption and reduce the emission of harmful pollutants. The popularization and promotion of electric vehicles (EVs) in the automotive industry have received widespread attention worldwide. Lithium-ion batteries (LIBs) have the characteristics of high energy density, high power density, long service life, environmental protection, and low self-discharge rate. It have become the preferred energy storage system for many applications such as EVs, grid energy storage, and other consumer electronics products^1,2^. However, the safety and reliable operating range of LIBs is very narrow, requiring a Battery Management System (BMS) to detect the voltage, current, and temperature of the battery pack, predict the charging and health status of the LIBs packs^3,4^, and effectively control, protect, and manage energy^5,6^. In addition, due to the limitations of battery voltage and storage capacity of individual LIBs, high-power applications of LIBs such as EVs and grid energy storage systems require hundreds or even thousands of LIBs to operate together, which will cause a common problem - single cell failure in LIBs packs. Therefore, a suitable battery fault diagnosis method is also essential for the safe and reliable operation of LIBs packs and each cell^7,8^.

In recent years, significant progress has been made in the research of fault diagnosis and prediction of LIBs. The faults in LIBs systems are usually divided into internal and external faults. Some of the most common external faults are battery connection failures, thermal management system failures, and sensor failures such as temperature, voltage, and current sensor failures, while some common internal battery faults are overcharging, over-discharging, internal short circuits, accelerated degradation, and thermal runaway^9^. Researchers have now recognized the importance of fault diagnosis for the safe and reliable operation of LIBs and conducted extensive research to develop accurate, reliable, robust, and easily implementable fault diagnosis strategies. Ref^10^. briefly explained why developing an effective fault diagnosis system is crucial for LIBs power supply systems. Ref^11,12^ discussed in detail the failure mechanism of LIBs and proposed possible solutions.

The fault diagnosis strategies reported in the literature can be roughly divided into model-based methods, knowledge-based methods, and data-driven methods. Among them, data-driven methods do not require establishing physical models of battery internal mechanisms. They directly distill sensitive features characterizing battery fault states from the physical signals collected to identify faulty batteries, which places high demands on data processing and modeling^13^. Among numerous data-driven algorithms, machine learning has been a hot research topic in recent years.

In order to identify faulty battery units and detect an early-stage internal short circuit fault, curvilinear Manhattan distances method to quantify the voltage changes of LIBs packs is proposed^14^. Ref^15^. proposes a method that combines fractional-order models and first-order resistance capacitance models. The model parameters are determined using genetic algorithms, and equivalent series resistance faults in batteries are identified through a random forest classifier, especially leakage related to external short circuits. A specialized transformer-based network architecture, which only uses time-resolved battery data as an input to estimate SOC is established^16^, The experimental results show that this method has good predictive ability^17^. A novel optimized multi-head attention method is proposed to compute multiple sets of LIBs within a network node in a simultaneous manner^18^. Naha et al.^19^ further developed a random forest based model for the online detection of internal short circuits, achieving a fault diagnosis accuracy of 97% by designing extreme conditions to generate training data. Yao et al.^20^ proposed an SVM diagnostic scheme that combines discrete cosine filtering and grid search to improve the prediction accuracy of series connected faults. Although the accuracy reaches 95%, the state recognition process is time-consuming, limiting online applications, and does not consider the effects of battery aging and temperature changes. Ref^21,22^ combined SVM with Gaussian process regression to develop a multi-model adaptive prediction method for detecting overcharge and under-discharge faults. Xue et al.^23^ developed a method based on statistical distribution, which effectively detects and locates abnormal units by real-time monitoring of battery pack status.

However, the above methods generally have some problems, such as complex data preprocessing, difficult parameter settings, and poor model adaptability. To address these issues, Artificial Intelligence (AI) methods especially Neural Network (NN) or Deep Learning (DL) with superior generalization capabilities has application prospects and significant advantages.

Ref^24^. develops a rapid multi-fault diagnosis method for the LIBs pack based on curvilinear Manhattan distance and voltage difference analysis technique. The proposed approach can sensitively and reliably detect and isolate multiple faults. Zhang et al.^25^ proposes a multi-task learning (MTL) framework based on a Convolutional neural network-Multi-gate Mixture of Gated recurrent units (CMMOG), which is capable of concurrently managing multiple SOH estimation regression tasks. Chen et al.^26^ proposes a LIBs equivalent circuit model and a DL network, and designs an improved Vision Transformer network (VIT), yielding a compete framework for predicting the SOH of LIBs. Due to the strong nonlinearity of battery degradation and complex working conditions, Ref^27^. developed a hybrid NN model with attentional mechanisms to achieve SOH estimation for LIBs. The developed model is composed of Convolution Neural Network (CNN), Convolutional Block Attention Module (CBAM), and Long Short-Term Memory (LSTM) NN. The experimental results show that the algorithm has a low estimation error. Similar research that a combining algorithms includes CNN, Variant LSTM (VLSTM) and Dimensional Attention mechanism (CNN-VLSTM-DA) also demonstrated the effectiveness^28^. Yan et al.^29^ used a nonlinear autoregressive exogenous NN to predict battery voltage and achieved early warning and multiple fault diagnosis through box plots, demonstrating the applicability and robustness of the model at different temperatures. Yao et al.^30^ proposed a fault diagnosis method based on CNN, which cleans voltage data through an empirical mode decomposition algorithm and expands the sample size using the sliding window method to ensure high accuracy. Ref^31^. combined attention mechanisms and domain adaptive NN to diagnose multiple types of faults, and the experimental results showed significant improvement under different conditions. Xu et al.^32^ developed an observer based on adaptive NN, which can adjust weights online and predict soft short circuit faults, demonstrating superiority. He et al.^33^ proposed a fault diagnosis method that combines physical models with DL, using LSTM networks to predict aging states, improving the accuracy of fault warning and demonstrating good practical application potential. Ref^34^. embedded an equivalent circuit model into a NN, combining the high precision of physical models with the powerful nonlinear processing capability of NN, greatly improving the effectiveness of fault diagnosis. Zhang et al.^35^ developed a DL framework for battery anomaly detection, which is easy to deploy in real-world environments and can effectively reduce the cost of fault detection. The DL framework proposed by Lee et al.^36^ is used to detect the reliability of sensor data, enhancing the safety and reliability of battery energy storage systems. Ref^37^. achieved accurate diagnosis of various faults in the LIBs pack by constructing an unsupervised learning model that combining CNN, convolutional LSTM network, and autoencoder.

In Ref^13^. , Zhang et al. proposed a novel graph-guided fault detection method designed to recognize concealed anomalies in realistic data. The method establish the coupling relationship and evolution of physical quantities under both normal and faulty states, effectively uncovering fault information hidden in collected battery data without observable anomalies.

Except for Ref^13^. , the existing methods are usually designed for a single fault, but in complex situations where multiple faults occur simultaneously and their causes are coupled, it is impossible to provide a unique diagnostic strategy for each type of fault. In response to the problems existing in machine learning based fault diagnosis technology, combined with the mutual coupling between faults in LIBs packs, the node-edge mechanism in Graphical Neural Networks (GNN) can better handle decoupling problems. Therefore, this article uses GNN to diagnose and analyze voltage faults in LIBs.

The main contributions of this article can be summarized as follows:

1) In order to handle the mutual coupling of multiple faults in LIBs packs diagnosis, the node-edge mechanism in GNN is introduced. At present, there are few research papers on the use of GNN for fault diagnosis of LIBs in EVs. This article focuses on the research of fault localization in fault diagnosis, and will further discuss fault prediction in the future.

2) In GraphSAGE with a single-layer architecture, setting the sampling layer to two can expand the information coverage range and improve performance, thereby avoiding the nonlinearity and computational overhead caused by multi-layer architecture.

3) The proposed method achieves a maximum improvement of 4.31% in the accuracy of abrupt fault localization and 3.68% in the accuracy of gradual fault localization through compared to the highest-performing baseline method.

The remainder of this paper is organized as follows. Section "Graphic neural network model" describes the principles of the GNN model. Section “Data preprocessing” describes the data preprocessing. Section "Optimized GNN Models for Fault Localization" proposed optimized GNN models for fault localization. The experimental results are discussed in Sect. “Experimental Results”. Finally, conclusions are drawn in Sect. “Conclusions”.

## Graphic neural network model

In the field of fault diagnosis and prediction, traditional deep neural network (DNN) methods typically focus on extracting unidimensional features from time series signals, while lacking systematic modeling of the spatial correlations between samples or multi-sensors^38^. To address the construction of spatial features, some studies have attempted to introduce CNN to capture local spatial patterns^39–41^. However, CNN are limited by their translation invariance assumption and fixed convolutional kernel design, making it difficult to explicitly represent the dynamic coupling relationships between samples or sensor nodes in non-Euclidean spaces. In contrast, GNN leverage a message-passing paradigm driven by topology structures, allowing adaptive aggregation of neighboring node state information and reconstruction of the physical dependency strength between samples or multi-sensors based on edge weights. This mechanism not only makes implicit relationships across nodes explicit, but also captures the cascading effects from local anomalies to global failures through hierarchical feature propagation. In this section, the basic concepts of graphs will be outline and the adaptability to battery systems will be discussed.

### Differences in GNN

**Fig. 1:**
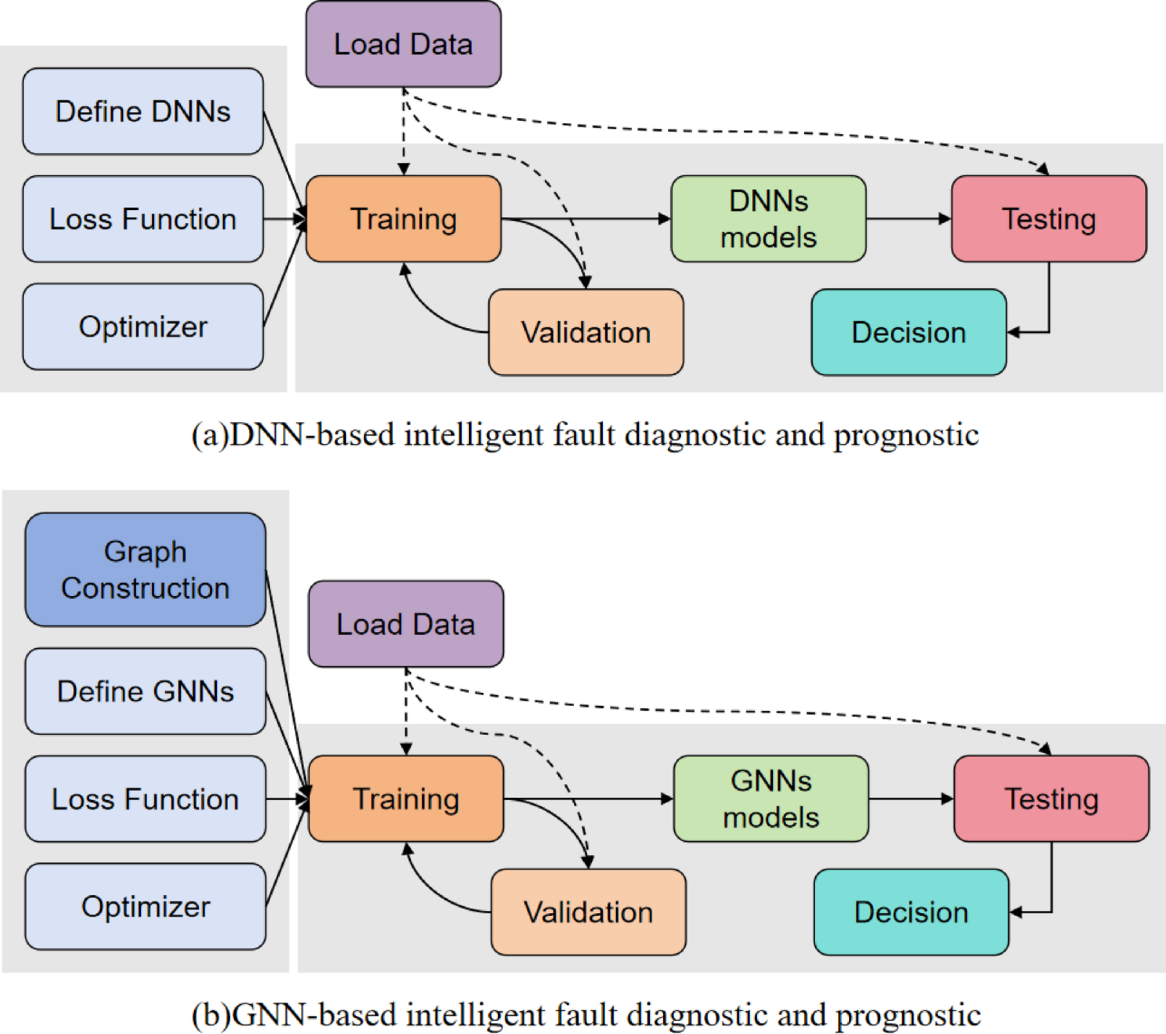
The framework of DNN-based methods and GNN-based methods.

Currently, the standard framework for DNN-based intelligent fault diagnosis and prediction is show in Fig. 1(a), it typically consists of four core components: data acquisition, model architecture design, parameters optimization training, and decision output^42^. In the data acquisition phase, the system collects raw operational data through sensors and other devices, then preprocesses and extracts features from the data, and ultimately segments it into sub-samples. Subsequently, based on the characteristics of the specific diagnostic task (such as the complexity of fault types, feature dimensions, etc.).

A DNN model with adaptive inter-layer connection structures and activation functions is constructed. During the model training process, the gradient backward propagation algorithm is employed to iteratively optimize the network parameters, enabling the model to effectively extract fault features from the training set by minimizing a predefined loss function. Once the model achieves the predetermined performance metrics through test set evaluation, the system can make decisions on new samples. Despite the significant advantages of DNN in deep feature representation of conventional data types (such as images, time series signals), the existing methods generally have the limitation of insufficient multi-source information fusion. Specifically, most models only focus on local feature extraction of a single sensor and fail to fully consider the spatiotemporal correlation characteristics between cross-modal data (such as sensor network topology, physical field coupling relationships). This modeling defect directly restricts the generalization ability of diagnostic models under complex working conditions. The main difference between GNN-based methods and others lies in the graph structure and the respective model designs, as shown in Fig. 1(b). Therefore, how to transform raw data into a graph structure and design an appropriate network model are two key challenges faced by GNN-based approaches.

**Fig. 2:**
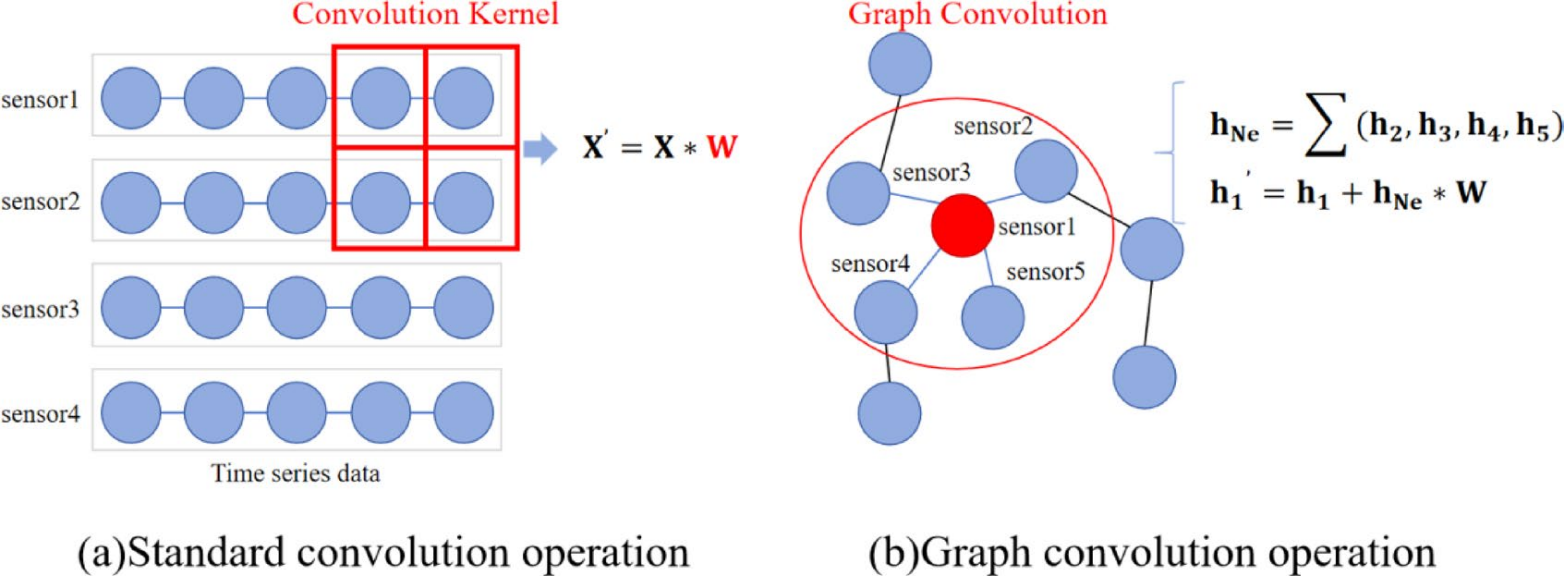
An illustration of convolution operation and graph convolution operation. (**a**) The results of the standard convolution operation are the sum of the point-wise multiplications of a subset of the signal $\:\mathbf{X}$ with a learned convolution filter $\:\mathbf{W}$. (**b**) The results of graph convolution operation could be the sum of the node feature itself with the product of the features of adjacent nodes $\:{\mathbf{h}}_{\mathbf{N}\mathbf{e}}$ and the learnable parameter $\:\mathbf{W}$.

In DNN/CNN architectures, standard convolutional kernels perform feature encoding on sensor data through a sliding window mechanism, as shown in Fig. 2(a). Essentially, this involves a linear weighted summation of spatiotemporal data from multiple sensor measurement points, but this approach has two significant limitations: firstly, the local receptive field design of convolutional kernels focuses only on the local neighborhood features of measurement points, neglecting the inherent topological connectivity of the sensor network. Secondly, conventional convolution operations assume spatial translation invariance among measurement points, making it impossible to model the physical coupling mechanisms between sensors in real industrial systems. To overcome this bottleneck, an increasing number of studies consider the interdependencies between data and model multi-source sensing data as graph-structured data^43,44^, as illustrated in Fig. 2(b). Under the framework of irregular graph data, dynamic interaction features between sensor nodes are quantitatively represented by edge weight matrices, where edge weights can reflect both physical connection strength and abstract relationships such as signal correlation. Unlike the fixed neighborhood structure in regular grid data, irregular graph data allow each node to establish dynamic neighborhood connections based on actual working conditions, making this feature representation more aligned with the characteristics of complex industrial systems.

### Compatibility of GNN with battery systems

**Fig. 3:**
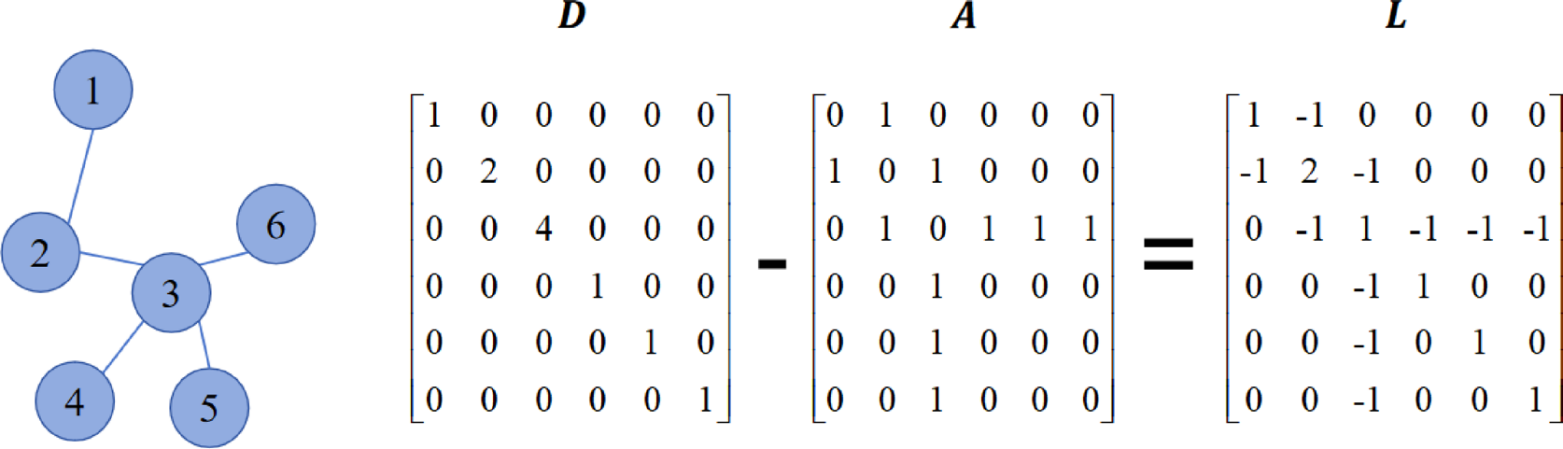
The calculation of Laplacian matrix.

In the theoretical framework of graph signal processing^45^, the graph structure can be formally defined as a triplet $\:G=(\varvec{X},\:\varvec{A},\:\varvec{E})$, whose mathematical representation includes the following core elements: the node feature matrix $\:\varvec{X}\in\:{\mathbb{R}}^{n\times\:d}$ represents a set of $\:d$-dimensional feature vectors of $\:n$ nodes, the edge set *E* defines the connection relationships between nodes, and the adjacency matrix $\:\varvec{A}\in\:{\mathbb{R}}^{n\times\:n}$ quantifies the connection strength between nodes $\:{v}_{i}$ and $\:{v}_{j}$ through elements $\:{A}_{ij}=({v}_{i},{v}_{j})\in\:\varvec{E}$ (as shown in Fig. 3). For undirected graphs, the adjacency matrix satisfies the symmetry condition $\:{A}_{ij}={A}_{ji}$; for directed graphs, $\:{A}_{ij}\ne\:{A}_{ji}$ is allowed to represent asymmetric connection relationships. Furthermore, the graph structure can be algebraically characterized by the following matrix forms:

1) Degree matrix $\:\varvec{D}\in\:{\mathbb{R}}^{n\times\:n}$: a diagonal matrix where the element $\:{D}_{ii}$ represents the connectivity strength of node $\:{v}_{i}$, and $\:\varvec{D}$ can be expressed as:

1 $$\:{D}_{ii}=\sum\:_{j}{A}_{ij}$$

2) Laplacian matrix $\:\varvec{L}\in\:{\mathbb{R}}^{n\times\:n}$: satisfying the positive semi-definite condition, defined as:

2 $$\:\varvec{L}=\varvec{D}-\varvec{A}$$

3) Symmetrically normalized Laplacian matrix: $\:\varvec{L}\_sym$used for spectral graph convolution operations, which can be expressed as:

3 $$\:\varvec{L}\_sym={\varvec{D}}^{-1/2}\varvec{L}{\varvec{D}}^{-1/2}$$

The description of the above matrices is shown in Fig. 3, which visually shows the irregular characteristics of the graph data.

The theoretical foundation of graph signal processing provides a rigorous mathematical framework for modeling complex systems, such as battery packs in EVs. A graph structure is formally defined as a triplet $\:G=(\varvec{X},\:\varvec{A},\:\varvec{E})$, where:

1) Node feature matrix $\:\varvec{X}\in\:{\mathbb{R}}^{n\times\:d}$ represents the $\:d$-dimensional feature vectors (such as voltage, temperature, impedance) of $\:n$ battery cells.

2) Edge set $\:\varvec{E}$ defines the physical or logical interactions between nodes.

3) Adjacency matrix $\:\varvec{A}\in\:{\mathbb{R}}^{n\times\:n}$ quantifies the interaction strength between nodes $\:{v}_{i}$ and $\:{v}_{j}$, with $\:{A}_{ij}=({v}_{i},{v}_{j})\in\:\varvec{E}$. For undirected graphs (such as symmetric thermal coupling), $\:{A}_{ij}={A}_{ji}$; for directed graphs (such as unidirectional current flow), $\:{A}_{ij}\ne\:{A}_{ji}$.

4) The diagonal elements $\:{D}_{ii}$ of the degree matrix $\:\varvec{D}\in\:{\mathbb{R}}^{n\times\:n}$ reflect the connection strength of nodes (such as battery cells with high connectivity potentially being at the core of heat exchange).

During the charging and discharging process of the battery pack, the data monitored by the sensors exhibits complex correlations due to physical coupling. The normal state system is modeled as a graph $\:G=(V,A)$, and the fault state as $\:{G}_{f}=({V}_{f},{A}_{f})$. The principle of graph-based fault diagnosis can be expressed through^46^

4 $$\:\{G=(V,A\left)\right\}\ne\:\{{G}_{f}=({V}_{f},{A}_{f}\left)\right\}$$

where $\:{V}_{f}$ and $\:{A}_{f}$ denote the feature matrix and the adjacency matrix in the faulty case, respectively. Specifically, if a fault occurs in a system, it may cause node feature deviation ($\:V\ne\:{V}_{f}$, $\:A={A}_{f}$), or lead to changes in the topological structure ($\:V={V}_{f}$, $\:A\ne\:{A}_{f}$), or both ($\:V\ne\:{V}_{f}$, $\:A\ne\:{A}_{f}$).

**Fig. 4:**
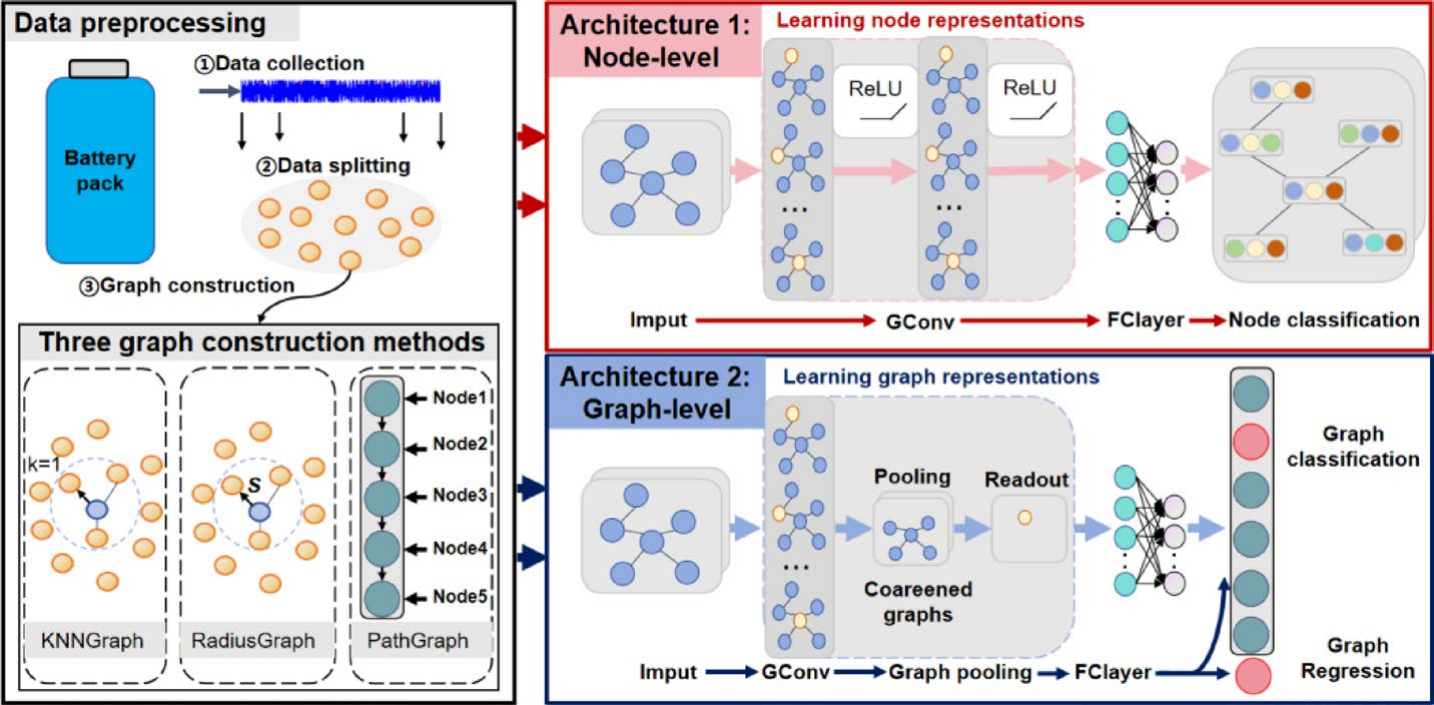
Fault diagnosis framework based on GNN.

The relational inductive bias of GNN naturally adapts it to such dynamic systems. As shown in Fig. 4, the GNN-based fault diagnosis framework consists of two core components:

1) Graph construction, encoding raw sensor data into a graph structure that preserves physical dependencies.

2) Model design, utilize message-passing mechanisms or spectral convolution to learn hierarchical representations of node-edge interactions.

As highlighted in the study^47^ provides a comprehensive guideline for implementing GNN in fault diagnosis, emphasizing the importance of graph construction and model design.

In summary, the mathematical formalism of graph signal processing provides the theoretical underpinning for modeling battery systems as dynamic graphs, while GNN offer the algorithmic tools to exploit these structures for robust fault diagnosis^48^. This synergy positions GNN as a promising paradigm for addressing the complexities of next-generation BMS.

## Data preprocessing

### Dataset introduction

In this paper, the MIT dataset is used to train and validate the performance of fault detection results. The MIT dataset consists of three LIBs (Packs #1–3), each containing approximately 46 cells, and it is publicly released by the Toyota Research Institute^49^. All the batteries in this dataset use either a one-step or two-step fast charging strategy, with slight variations in the charging strategy for different cells within the same pack. This leads to varying cycle lifetimes, ranging from 150 to 2300 cycles. The cycle life is defined as the number of cycles until the battery reaches 80% of its nominal capacity. The sampling frequency of the MIT dataset is 0.18 Hz, and the upper and lower cut-off voltages are 3.6 V and 2.0 V, respectively. The battery parameters are shown in Table 1.

**Table 1:** Battery parameters of MIT dataset.

Parameter type	MIT dataset
Temperature	30℃
Rated capacity	1.1Ah
Charge cut-off voltage	3.6 V
Discharge cut-off voltage	2.0 V
Discharge rate	4 C
Sampling frequency	0.18 Hz

Given the MIT dataset comprising multiple battery packs (Pack #1–3, each with approximately 46 cells), its inherent cell heterogeneity provides an ideal foundation for generating diverse fault samples. Based on the fault mode classification of LIBs in reference^50^, the study focuses on two typical scenarios: abrupt faults and slow faults. Abrupt faults are usually caused by intense sensor interference or battery short circuits, while slow faults are caused by natural battery aging and improper operations, such as overcharging, over-discharging, and electrolyte leakage. Inspired by the hybrid fault injection methodology proposed in^51^, the study adopts a phased controllable injection strategy. In abrupt fault diagnosis, all cells of the first cycle are used for abrupt fault injection, while in slow fault diagnosis, all cells of the last cycle are used for slow fault injection.

**Fig. 5:**
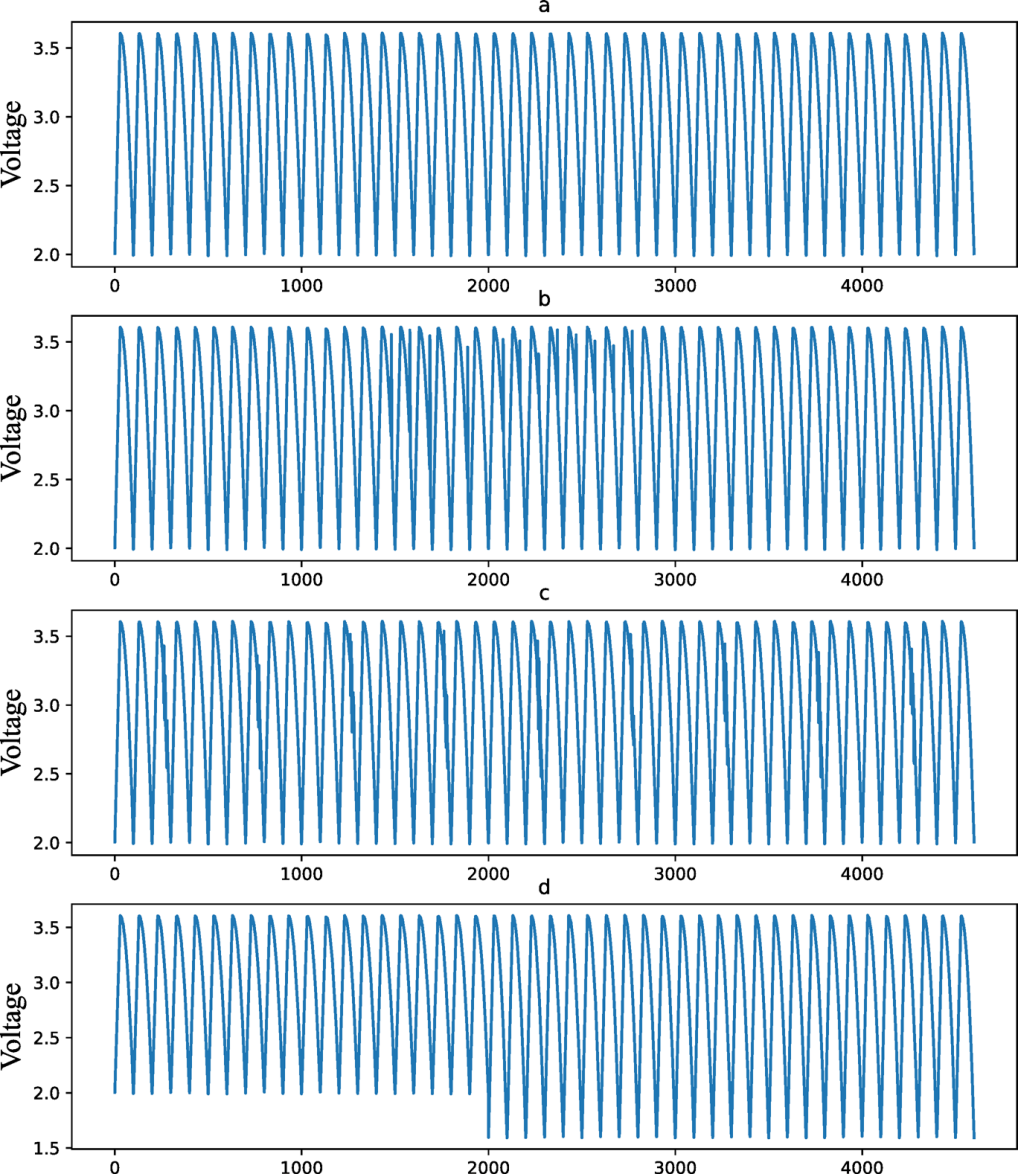
Examples of different voltage samples in the MIT dataset. (**a**) Normal. (**b**) Sudden voltage changes. (**c**) Random voltage fluctuations. (**d**) Slow faults.

Abrupt fault injection includes two forms: sudden voltage changes (as shown in Fig. 5(b), voltage step mutations (± 10%~20% of rated voltage) to emulate sensor failures) and random voltage fluctuations (as shown in Fig. 5(c), additive Gaussian white noise to simulate random disturbances). Slow fault injection involves a reduction in the discharge cut-off voltage (as shown in Fig. 5(d), the voltage is continuously out of the normal range for a long time). The normal and fault samples of the MIT dataset are illustrated in Fig. 5.

### Fault injection

Gu et al.^52^ reference mentions that during a abrupt voltage fault, the voltage curve deviates from the normal trend, showing a sudden increase or sharp decrease, which leads to significant local variance differences. The present study performs data preprocessing using a sliding window approach, followed by fault injection to achieve real-time fault localization. The data preprocessing follows three steps:

(1) Set different sliding window sizes (ranging from 3 to 6), moving forward one time step at a time. At each step, the voltage data from all batteries within the window are recorded and combined into a single sample. This process generates a series of time-ordered voltage data samples. The windowed data not only preserves the temporal characteristics of the data but also allows for better analysis of local data, reducing computational complexity and facilitating practical deployment.

(2) For abrupt faults, in contrast to^52^, a more intuitive local standard deviation has been chosen and significant variation as the criterion for fault injection has been used. The formula for calculating the local standard deviation based on the sliding window is as follows:

5 $$\:{\text{S}\text{D}}_{\text{i},\text{p}}=\sqrt{\frac{\sum\:_{\text{t}=\text{p}-{\upomega\:}}^{\text{p}}{({\text{V}}_{\text{i},\text{p}}-{\overline{\text{V}}}_{\text{i},\text{p}})}^{2}}{{\upomega\:}}}$$

where $\:{\upomega\:}$ denotes the sliding window width; $\:{\overline{\text{V}}}_{\text{i},\text{p}}$ denotes the mean value of cell $\:\text{i}$ in the $\:\text{p}$-th sliding window; and $\:{\text{S}\text{D}}_{\text{i},\text{p}}$ denotes the local variance in the $\:\text{p}$-th sliding window.

The control limit serves as the threshold for determining whether the process is normal and plays a key role in fault detection^53,54^. The control limit determination method adopted in this study is an optimized design based on the traditional empirical approach. Compared with statistical inference methods based on Gaussian distribution assumptions, the empirical method not only avoids the limitations of distributional assumptions but also demonstrates stronger adaptability in real-world engineering scenarios. Given that the detection index (with the standard deviation studied in this work) exhibits significant irregular characteristics, a non-parametric empirical distribution method is used to construct the control limit. Based on a significance level of $\:{\upalpha\:}=0.01$, the final control limit is set to cover 99% of the detection index distribution in the training set. This threshold setting not only complies with the normative requirements of statistical hypothesis testing but also effectively balances the engineering demands between false alarm rate and detection sensitivity. The formula for calculating the threshold is as follows:

6 $$\:{\text{S}\text{D}}_{\text{I},\text{P}}=\left\{{\text{S}\text{D}}_{\text{i},\text{p}}|\text{i}=\text{1,2},...\text{M},\:\text{p}=\text{1,2},...\text{N}\right\}$$

7 $$\:T\text{h}\text{r}\text{e}\text{s}\text{h}\text{o}\text{l}\text{d}=\text{P}\text{e}\text{r}\text{c}\text{e}\text{n}\text{t}\text{i}\text{l}\text{e}({\text{S}\text{D}}_{\text{I},\text{P}},\:99)$$

where $\:\text{M}$ and $\:\text{N}$ represent the number of batteries in the battery pack and the number of sliding windows for each battery, respectively; $\:{\text{S}\text{D}}_{\text{I},\text{P}}$ denotes the set of all local voltage standard deviations in the battery pack; and $\:T\text{h}\text{r}\text{e}\text{s}\text{h}\text{o}\text{l}\text{d}$ represents the abrupt fault threshold of the battery pack.

**Fig. 6:**
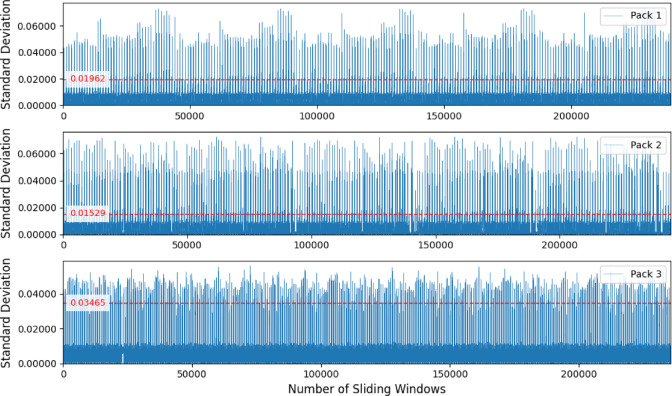
The standard deviation of the data collected with a window size of three.

Figure 6 shows the threshold selection for each battery pack when the window size is three, it can be seen that the voltage differences between different battery packs are still quite obvious.

For slow faults, unlike abrupt faults, there is no need to set a threshold for fault injection. The voltage data from the last charge-discharge cycle of the same battery can be considered as fault data for replacement, which aligns with the signal changes that occur during the onset of slow faults.

(3) Due to differences among the individual cells in the battery pack, the voltage time series data during charging and discharging have varying lengths. We selected the shortest time series among the individual cells in each battery pack as the time series for the entire pack. Taking the first cycle of charge and discharge data from each battery pack as an example, the trimmed data are as follows:

1) Battery pack 1 has 46 cells, with a voltage time series length of 1041.

2) Battery pack 2 has 48 cells, with a voltage time series length of 1034.

3) Battery pack 3 has 46 cells, with a voltage time series length of 734.

On this basis, fault injection is performed according to the sliding window size to form a fault localization dataset with a ratio of 1:10 between fault data and normal data. The dataset is split into training, validation, and test sets in a ratio of 6:2:2.

## Optimized GNN models for fault localization

The proposed method takes into account that the number of normal segments far exceeds the number of fault segments in real battery datasets. This imbalance causes many models to favor normal samples during training, leading to false positives in locating faulty batteries. To address this issue, this study proposes a GraphSAGE model based on adaptive graph structure optimization, using the fault injection dataset described in Sect. 3, for locating faulty batteries in the systems.

### Dynamic correlation-based graph constructor

This study proposes a graph structure representation framework based on the heterogeneity of coupling relationships between battery cells to construct a physical topology graph. Its theoretical basis is reflected in the following two aspects:

Firstly, under complex working conditions, the battery system not only exists explicit electrical connections but also contains multi-physics coupling effects composed of factors such as temperature field distribution and aging state transmission. Traditional graph construction methods often establish adjacency matrices based on the isotropic assumption, ignoring the modulating effect of connection strength on information propagation, leading to deviations between topology representation and physical reality.

Secondly, although the fully connected graph structure can theoretically retain all potential associations, it will actually introduce topology redundancy noise. In response, this study designs a neighborhood selection mechanism based on correlation strength: by introducing the node correlation coefficient, sparse processing is performed on weakly associated edges before training. This mechanism ensures the integrity of key physical associations while reducing the computational complexity of adjacency matrix calculation.

Specifically, according to Eq. (8), the Pearson correlation coefficient matrix between the battery cells in the training set is calculated to quantify the coupling strength of the battery cells in their operating state, and avoid the insufficiency of the traditional adjacency matrix that can only represent whether there is a connection. To mitigate the impact of low-relevance information in the topology, a threshold filtering mechanism is introduced: the selection of the threshold is similar to a hyperparameter, and 0.6 is determined as the optimal threshold through multiple rounds of training. The adjacency matrix constructed on this basis satisfies Eq. (9).

8 $$\:{\text{r}}_{\text{i}\text{j}}=\frac{{\sum\:}_{\text{k}=1}^{{\upomega\:}}{(\text{V}}_{\text{i},\text{k}}-{\overline{\text{V}}}_{\text{i}}){(\text{V}}_{\text{j},\text{k}}-{\overline{\text{V}}}_{\text{j}})}{\sqrt{{\sum\:}_{\text{k}=1}^{{\upomega\:}}{{(\text{V}}_{\text{i},\text{k}}-{\overline{\text{V}}}_{\text{i}})}^{2}}\sqrt{{\sum\:}_{\text{k}=1}^{{\upomega\:}}{{(\text{V}}_{\text{j},\text{k}}-{\overline{\text{V}}}_{\text{j}})}^{2}}}$$

9 $$\:\left\{\begin{array}{c}{\text{A}}_{\text{i}\text{j}}={\text{r}}_{\text{i}\text{j}},\:{\text{r}}_{\text{i}\text{j}}\ge\:0.6\\\:\:\:\:\:\:\:\:0\:\:\:\:\:\:,\:{\text{r}}_{\text{i}\text{j}}\le\:0.6\end{array}\right.$$

where $\:{\text{r}}_{\text{i}\text{j}}$ represents the Pearson correlation coefficient.

**Fig. 7:**
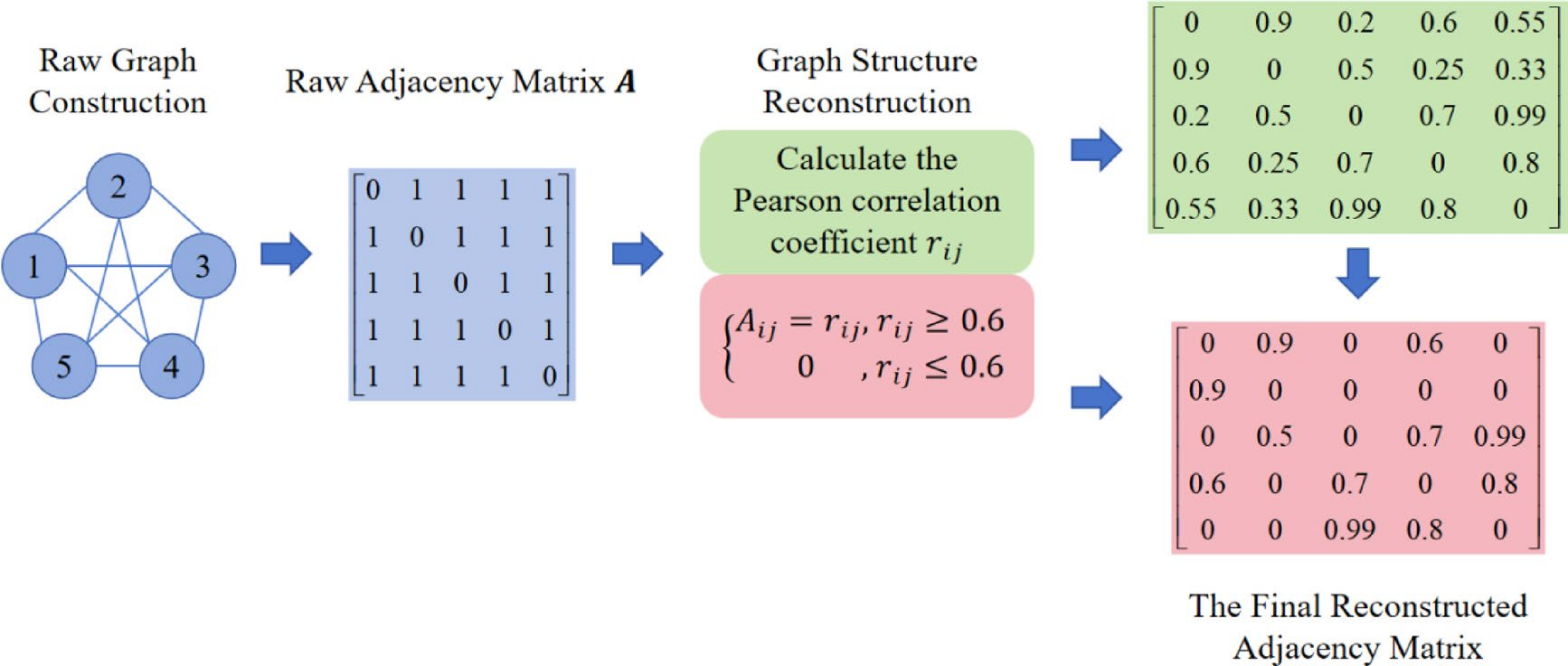
Schematic diagram of adjacency matrix reconstruction based on Pearson correlation coefficient.

This construction method offers dual advantages: firstly, it transforms implicit relationships like thermal-electric coupling into computable explicit topological connections through the Pearson correlation coefficient, enhancing physical consistency compared to traditional binary adjacency matrices. Secondly, the threshold mechanism increases the sparsity of the adjacency matrix, reducing redundant computations compared to fully connected graphs. As shown in Fig. 7, this approach effectively filters out spurious correlations caused by random noise while preserving the integrity of strongly correlated edges.

### Optimized GNN model

To accurately model the complex nonlinear correlation characteristics between battery voltage data and operational states, this study introduces the GNN GraphSAGE into the fault localization model. As a representative graph learning method, GraphSAGE generates embedding representations by aggregating neighborhood node features, effectively capturing the topological correlation characteristics among multidimensional data^55^. The core mechanism of this algorithm consists of two parts: the forward propagation of GraphSAGE and the backward propagation of GraphSAGE^56,57^.

**Fig. 8:**
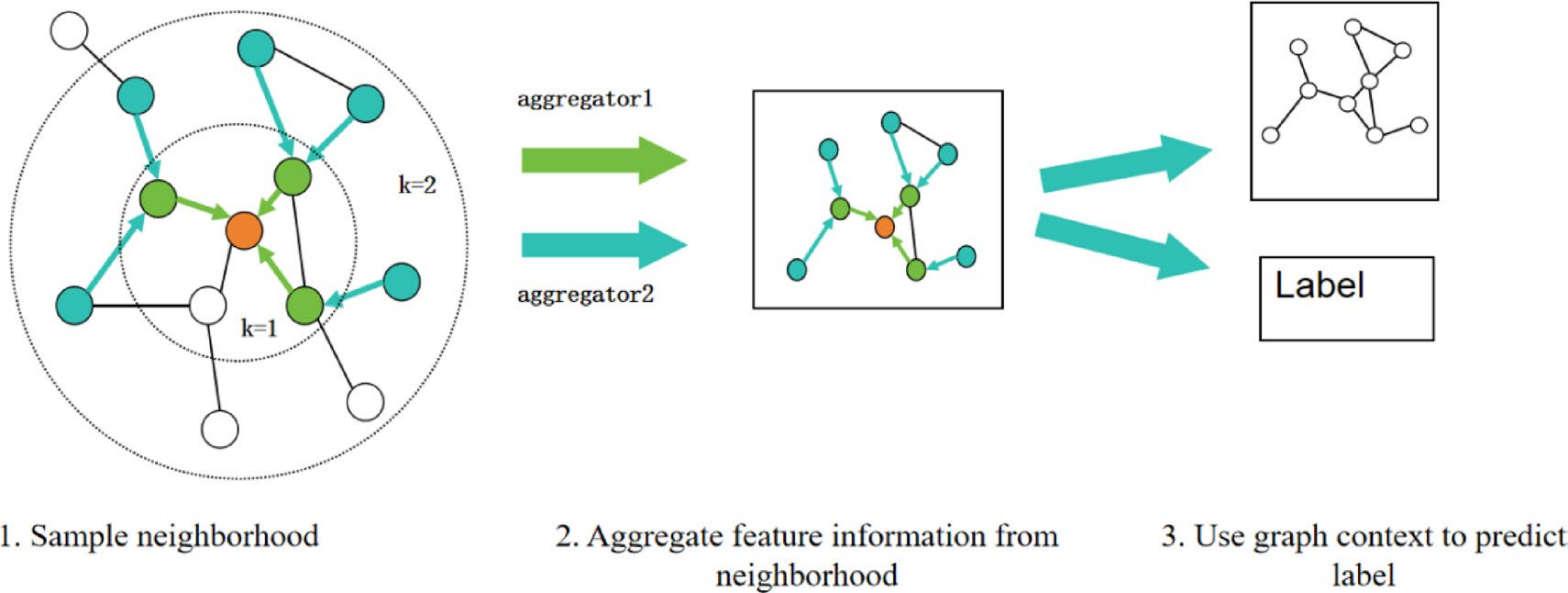
Visual illustration of the GraphSAGE sample and aggregate approach.

1) Forward propagation of GraphSAGE: The algorithm first constructs the local topological structure of the target node through a dynamic neighborhood sampling strategy. Subsequently, the network iteratively performs feature aggregation operations in a hierarchical manner to aggregate information contained in neighboring nodes, as shown in Fig. 8. Specifically, for node $\:\text{v}$ in the $\:\text{l}$-th layer of the NN, its representation vector $\:{\text{h}}_{\text{v}}^{\left(\text{l}\right)}$ is obtained by compressing the features of neighboring nodes from the previous layer $\:{\text{h}}_{\text{u}}^{(\text{l}-1)}$ through an aggregation function, followed by a linear transformation with a weight matrix and mapping via an activation function, as shown in Eq. (10). The final generated node embedding not only integrate the node’s own attributes and direct neighbor relationships but also capture dynamic patterns across nodes through multi-layer propagation mechanisms, forming a feature space with multi-scale perception capabilities. Ultimately, the $\:Readout$ function merges the final embedding representations of all nodes into the global representation of the graph, which is then fed into a dense layer to obtain the output probabilities, as shown in Eq. (11).

10 $$\:{h}_{v}^{\left(l\right)}=\sigma\:({W}^{\left(l\right)}\bullet\:AGGREGATE(\left\{{h}_{u}^{(l-1)}|\forall\:u\in\:N\left(v\right)\right\}\left)\right)$$

11 $$\:{\widehat{y}}_{v}=Dense\left(Readout\right(\left\{{h}_{v}^{\left(l\right)}|\forall\:v\in\:{\mathcal{V}}_{train}\right\}\left)\right)$$

where $\:AGGREGATE$ denotes the aggregation function of embedding of sampled neighboring nodes, $\:\text{N}\left(\text{v}\right)$ is the sampling neighborhood, $\:{\text{W}}^{\left(\text{l}\right)}$ is the trainable weight matrix, and $\:{\upsigma\:}$ is the nonlinear activation function. $\:{\widehat{\text{y}}}_{\text{v}}$ is the predicted probability distribution, $\:{\mathcal{V}}_{\text{t}\text{r}\text{a}\text{i}\text{n}}$ is the set of training nodes.

2) Backward propagation of GraphSAGE: Firstly, a crossentropy loss function is constructed based on the supervised learning paradigm, as shown in Eq. (12). The model error is quantified by calculating the difference between the model’s predicted fault probability distribution and the true labels. Subsequently, automatic differentiation technology is used to propagate gradient signals backward through the computational graph, employing the chain rule to compute the partial derivatives of the loss function with respect to the weight matrices of each layer and the parameters of the aggregation function. During this process, the network dynamically adjusts the parameter combinations of the feature aggregation layers and nonlinear transformation layers, gradually aligning the node embedding space with the fault pattern distribution characteristics of the battery system. Finally, the model parameters are iteratively updated by combining the adaptive learning rate mechanism of the optimizer, reducing the training loss while preserving parameter sharing properties, thereby ensuring the model can generalize to unseen battery topologies.

12 $$\:\mathcal{L}=-\sum\:_{v\in\:{\mathcal{V}}_{train}}{y}_{v}log{\widehat{y}}_{v}$$

where $\:\mathcal{L}$ is the model error, and $\:{y}_{v}$ is the true label.

**Fig. 9:**
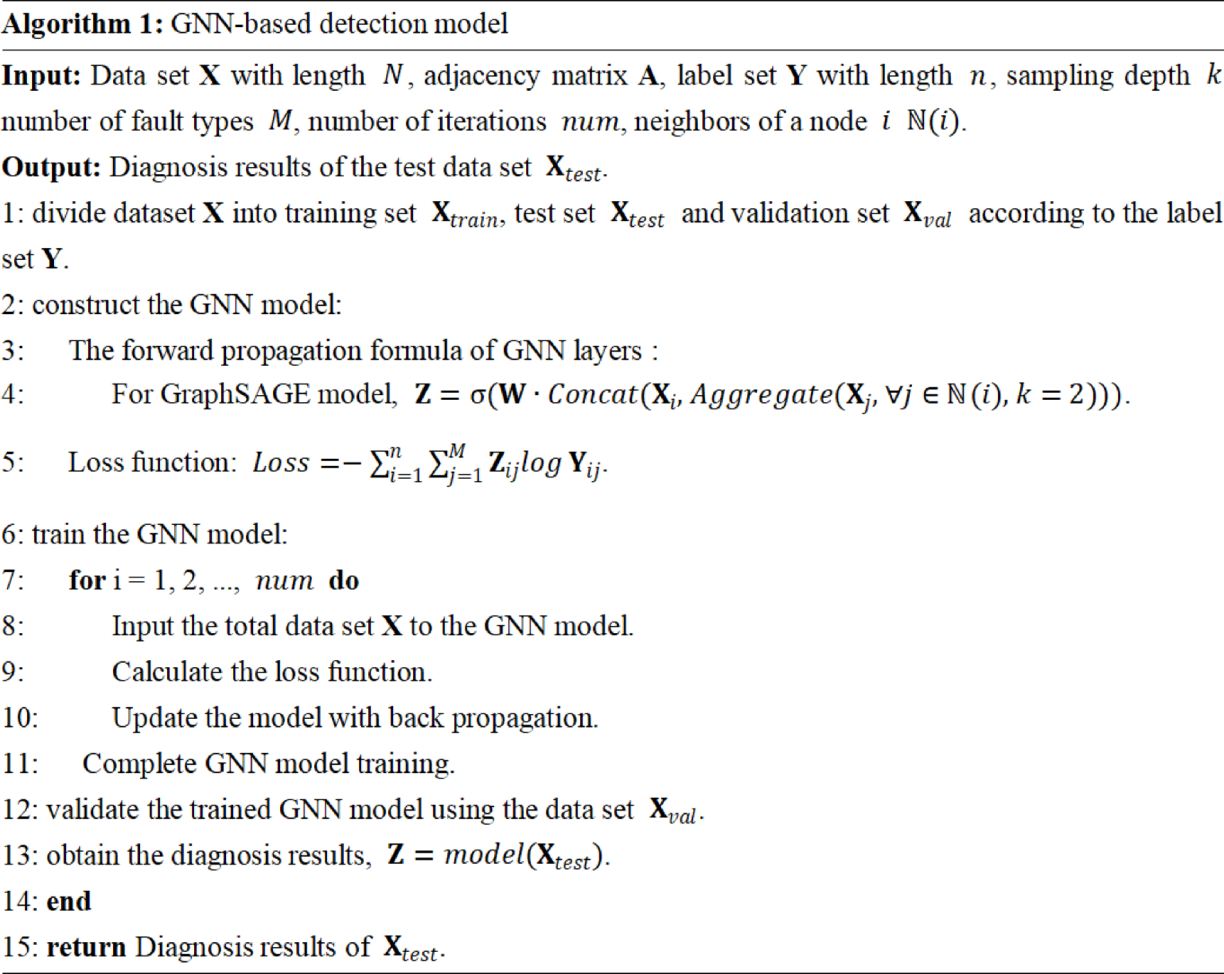
Pseudo code of GNN-based fault localization.

### Implementation details and model training

Based on the theoretical framework of forward propagation and backward propagation, the specific implementation of the model needs to integrate the actual data characteristics of the battery system. As shown in Fig. 9, the pseudocode clearly reflects the multi-layer aggregation mechanism and training process of GraphSAGE. Firstly, the input battery voltage data $\:(V,\:A)$ is transformed into graph-structured data through the graph construction module, where node features represent voltage time-series information, and edge weights are calculated based on physical connections or electrical correlations between battery cells. Subsequently, the model employs a two-layer GraphSAGE convolutional layer $\:(k=2)$, each followed by a GraphNorm layer to accelerate convergence, and enhances nonlinear expressive power through the ReLU activation function. To prevent overfitting, a Dropout layer is inserted between the two fully connected layers, and the final output layer generates a fault probability distribution via the Softmax function. Using cross-entropy as the loss function to evaluate the model’s accuracy, the Adam optimizer updates weights during backward propagation. Table 2 shows the optimal hyperparameters selected in this paper.

**Table 2:** Model parameters.

	Hyperparameters	Setting
General parameters	Epoch	300
	Optimizer	Adam
	Loss function	Cross-entropy
	Batch size	256
	Learning rate	0.001
GraphSAGE	Activation function	ReLU
	Number of layers	2
	Number of kernels	64
	Readout	Linear
Dense	Dropout rate	0.5
	Activation function	Softmax
	Number of layers	1
	Number of neurons	47–49

**Fig. 10:**
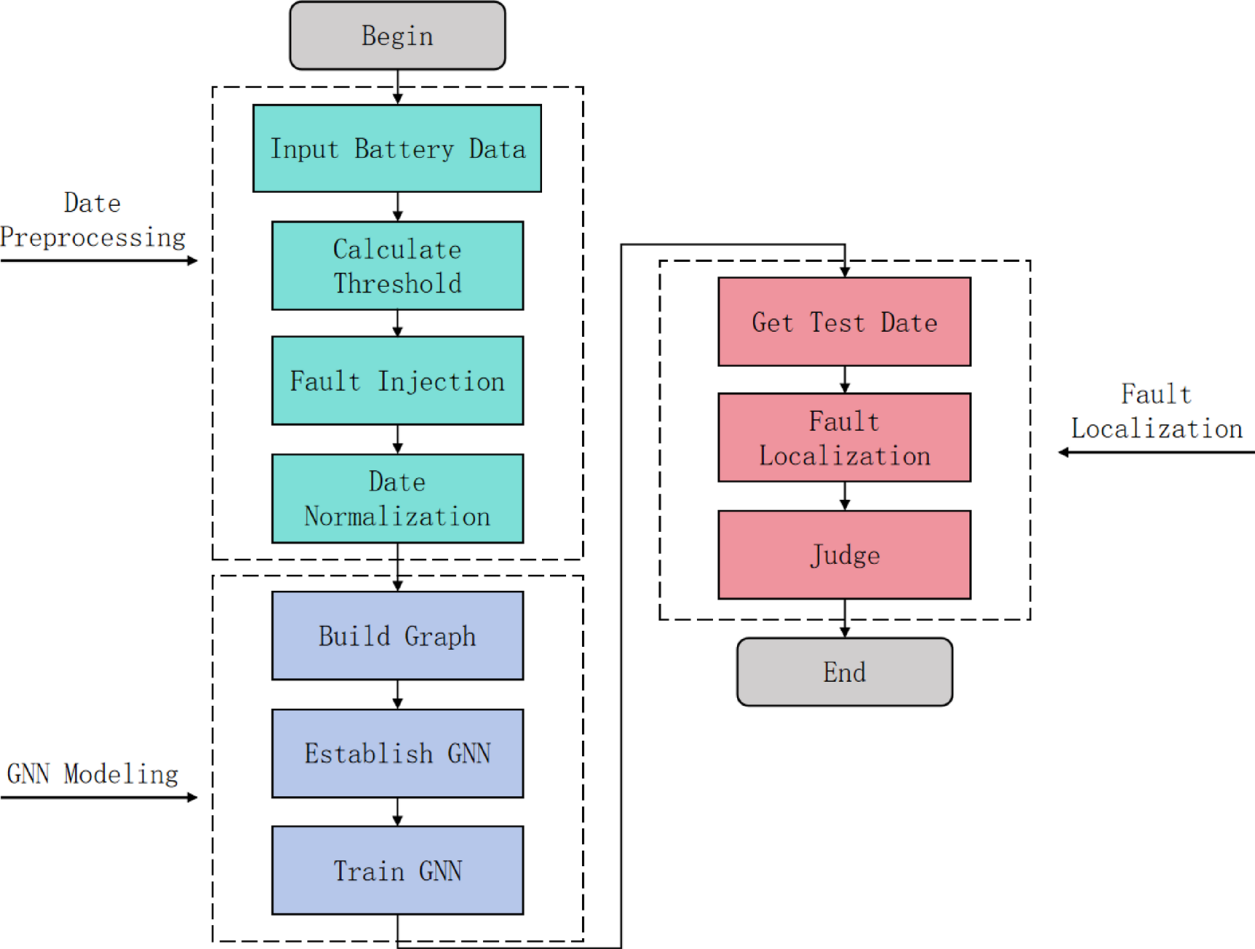
Framework of the fault localization model.

The flow chart of the fault localization model is shown in Fig. 10, the process begins with data preprocessing, which includes inputting battery data, calculating thresholds, injecting faults, and normalizing data. After data preprocessing, the workflow moves to the GNN modeling phase. This involves constructing the graph, building the GNN model, and training it. Once GNN modeling is completed, the workflow advances to the fault diagnosis phase, where test data is obtained, fault localization is conducted, and finally, a determination is made to identify the presence of any faults.

## Experimental results

In our proposed model, we apply an optimized GNN to the field of fault localization in EVs batteries. The programming environment for the experiments includes Windows 11 operating system, Python 3.9.20, and PyTorch 2.3.0. The experiments use the charge-discharge time series data of EVs batteries, with the feature names of the data kept hidden.

### Evaluation metrics

Fault localization can be understood as a multi-class classification problem, with categories divided into a normal class and multiple fault classes. If the dataset contains N batteries, fault classes are labeled as 1 to N, and the normal class is labeled as 0. Since the preprocessed battery dataset contains far more normal data than abnormal data, accuracy alone cannot serve as an evaluation metric as it does in typical classification problems. We use four evaluation metrics: accuracy, precision, recall, and F1-score. $\:TP\:$is true positive samples, $\:FP$ is false-positive samples, $\:FN$ is false negative samples, and $\:TN$ is true negative samples.

The accuracy is the proportion of $\:TP$ samples and $\:TN$ samples to all samples, which can be calculated as

13 $$\:Accuracy=\frac{TP+TN}{TP+TN+FP+FN}$$

The precision rate is the proportion of $\:TP$ samples to those predicted to be positive, which can be expressed by

14 $$\:Precision=\frac{TP}{TP+FP}$$

The recall is the proportion of $\:TP$ samples to those that are actually positive, which can be calculated as

15 $$\:Recall=\frac{TP}{TP+FN}$$

The F1-score is calculated from recall and precision and is a composite metric for anomaly localization. It can be expressed by

16 $$\:F1=\frac{2Recall*Precision}{Recall+Precision}=\frac{2TP}{2TP+FP+FN}$$

Note that the four metrics above, $\:Recall$, $\:Precision$, $\:Accuracy$, and $\:F1$, the bigger the better.

### Experimental setup

To verify the effectiveness of the GraphSAGE model in this paper, we compare the proposed model with baseline methods based on graph convolutional networks. The parameter settings of these network models are consistent with those of the proposed model in this paper. Specifically, we used the following baseline methods as comparisons.

1. GraphConv: A generalized graph convolution operation, typically referring to a common convolution method used in various GNN.
2. GCNConv: Kipf et al.^58^ proposed GCNConv, which introduced the basic principles of graph convolution based on spectral graph theory. It is one of the foundational methods in GCN.
3. SGConv: Wu et al.^59^ proposed SGConv, or simplified GCN, which significantly improves computational efficiency by removing the non-linear parts of GCN, making it particularly suitable for large-scale graph data.
4. ChebConv: Michaël et al.^60^ proposed a graph convolution operation based on Chebyshev polynomials, originating from the framework of spectral GCN. ChebConv performs convolution on graph signals in the frequency domain to capture the features of nodes and their neighbors.

In addition to comparison with the aforementioned different GNN, we also conducted a vertical comparison of the proposed model with classic methods, including CNN, DBN, LSTM and CNN-LSTM^61^.

In this experiment, all packs from the MIT dataset were used to train and evaluate the model. Additionally, the impact of different window sizes (ranging from 3 to 6) in the preprocessing of data on model performance was explored. Each input sequence consists of voltage data from three time steps. The number of nodes represents the number of features in the data, which corresponds to the number of batteries, while the number of neighboring nodes for each feature node indicates the weights of the neighboring nodes retained during graph construction. To ensure the effectiveness and fairness of the experiment, the parameters of the comparison algorithm models have been adjusted as much as possible to achieve optimal results in fault localization for the dataset used in this study. The loss function employs the Adam optimizer, with a learning rate set to 0.001. The activation function and other parameter settings are consistent, and the experimental environment is maintained uniformly. Note that when we conduct comparative experiments, we should try to set the receptive fields to be consistent as much as possible.

### Analysis of the experimental results

**Fig. 11:**
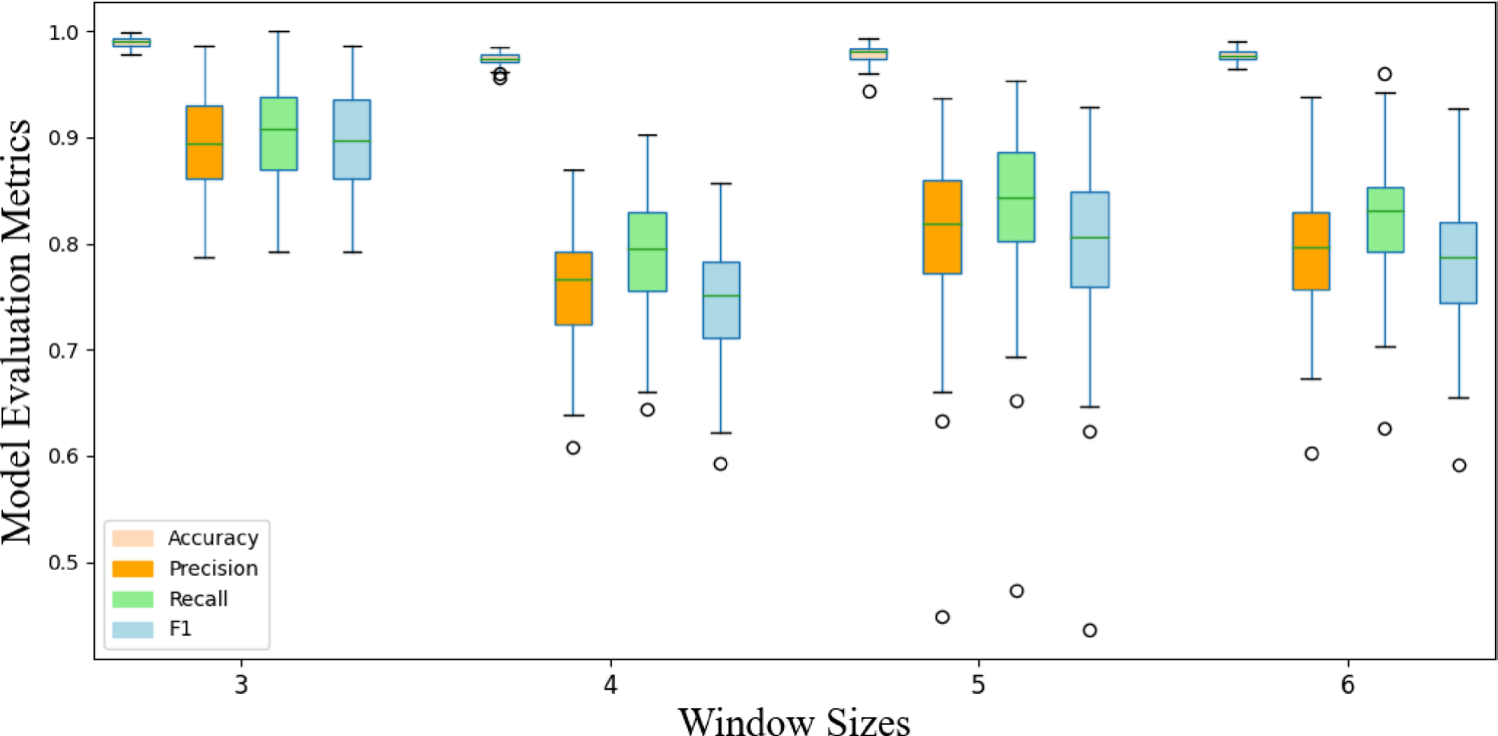
Impact of different window sizes on model training results.

The preprocessed data is fed into the GNN framework for model training. To evaluate the statistical stability of model performance, the study employs the Monte Carlo method to conduct 100 independent repeated experiments and constructs a performance fluctuation distribution graph. As shown in Fig. 11, by comparing the impact of different time window parameters on model convergence characteristics, it was found that when the sliding window size is set to 3, the model achieves an average F1-score of 0.895 on the validation set, significantly outperforming other parameter configurations. This phenomenon may stem from the symmetric structure of odd-numbered windows effectively preserving phase information in temporal features while suppressing high-frequency noise interference, thereby enhancing the separability of fault patterns in the feature space. Based on statistical significance test results, subsequent experiments will adopt a window size of 3 as the standardized preprocessing parameter.

Using the dataset preprocessed with a window size of 3, we perform a comparison of the various models. Tables 3 and 4 present the comparison of results for the best-performing models in abrupt fault diagnosis and slow fault diagnosis across 100 training iterations, respectively. It is evident that across the three battery pack datasets, our GraphSAGE outperforms other models on all four evaluation metrics. Moreover, by comparing Recall and F1-score, it can be seen that the learning effectiveness of GCNConv, GraphConv, and SGConv for fault samples is barely satisfactory. In pack 1, the accuracy for abrupt fault localization reaches as high as 99.90%, while for gradual fault localization, accuracy consistently exceeds 99%. Additionally, models trained with data from pack 1 demonstrates overall better performance. In the upcoming experiments, we will use the best-performing battery pack data to conduct comparative experiments with other NN models.

**Table 3:** The comparison of results for the best-performing models in abrupt fault.

Battery Packs	GCN	Accuracy	Precision	Recall	F1
Pack 1	GCNConv	0.9307	0.0872	0.0912	0.0891
	GraphConv	0.9326	0.0654	0.0700	0.0676
	SGConv	0.9307	0.0872	0.0912	0.0891
	ChebConv	0.9951	0.9442	0.9443	0.9442
	GraphSAGE (our model)	**0.9990**	**0.9868**	**0.9868**	**0.9868**
Pack 2	GCNConv	0.9323	0.0573	0.0614	0.0593
	GraphConv	0.931	0.0608	0.0653	0.0629
	SGConv	0.931	0.0689	0.0825	0.074
	ChebConv	0.9648	0.6562	0.6296	0.6387
	GraphSAGE (our model)	**0.974**	**0.7419**	**0.6535**	**0.6828**
Pack 3	GCNConv	0.9284	0.0597	0.0643	0.0619
	GraphConv	0.9323	0.0645	0.0691	0.0667
	SGConv	0.9297	0.0578	0.0618	0.0598
	ChebConv	0.9909	0.8469	0.8474	0.8471
	GraphSAGE (our model)	**0.9935**	**0.9312**	**0.9091**	**0.9164**

**Table 4:** The comparison of results for the best-performing models in slow fault.

Battery Packs	GCN	Accuracy	Precision	Recall	F1
Pack 1	GCNConv	0.9297	0.0549	0.0589	0.0568
	GraphConv	0.9297	0.0624	0.0667	0.0645
	SGConv	0.9307	0.0628	0.0674	0.065
	ChebConv	0.9961	0.9604	0.9624	0.9611
	GraphSAGE (our model)	**0.9971**	**0.9644**	**0.9635**	**0.9619**
Pack 2	GCNConv	0.9323	0.0578	0.0619	0.0598
	GraphConv	0.9414	0.4001	0.3776	0.3812
	SGConv	0.9271	0.0541	0.0582	0.0561
	ChebConv	**0.9974**	0.9455	0.9531	0.9481
	GraphSAGE (our model)	**0.9974**	**0.9493**	**0.9565**	**0.9517**
Pack 3	GCNConv	0.9414	0.0643	0.0683	0.0662
	GraphConv	0.9661	0.4962	0.4919	0.485
	SGConv	0.9414	0.082	0.0859	0.0838
	ChebConv	0.9922	0.8678	0.8722	0.8646
	GraphSAGE (our model)	**0.9935**	**0.8902**	**0.9008**	**0.8929**

**Fig. 12:**
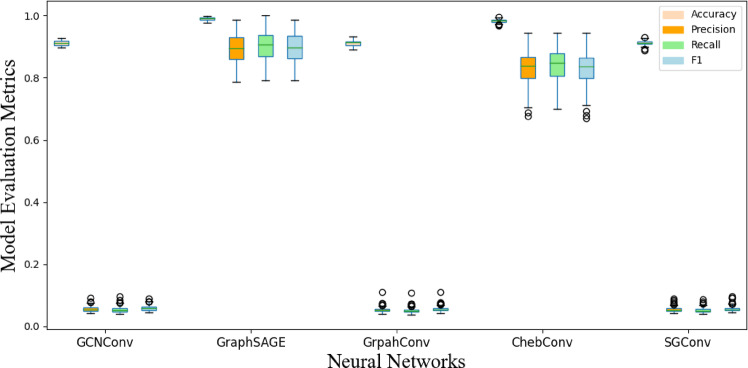
Performance comparison of different models for locating abrupt faults.

**Fig. 13:**
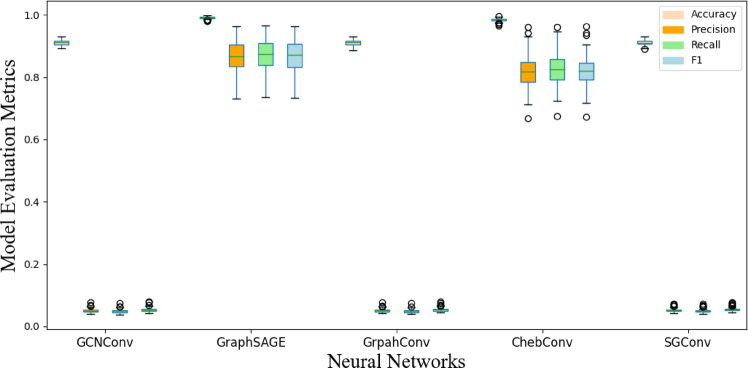
Performance comparison of different models for locating slow faults.

Figures 12 and 13 display the box plots of 100 training results, effectively presenting the distribution of the four evaluation metrics. It can be observed that, whether for abrupt or slow faults, GraphSAGE’s flexibility in sampling strategies and feature aggregation enables it to balance local mutations with global trends. Additionally, GraphSAGE’s metrics are more stable, indicating its higher tolerance to noise and sparse data.

**Fig. 14:**
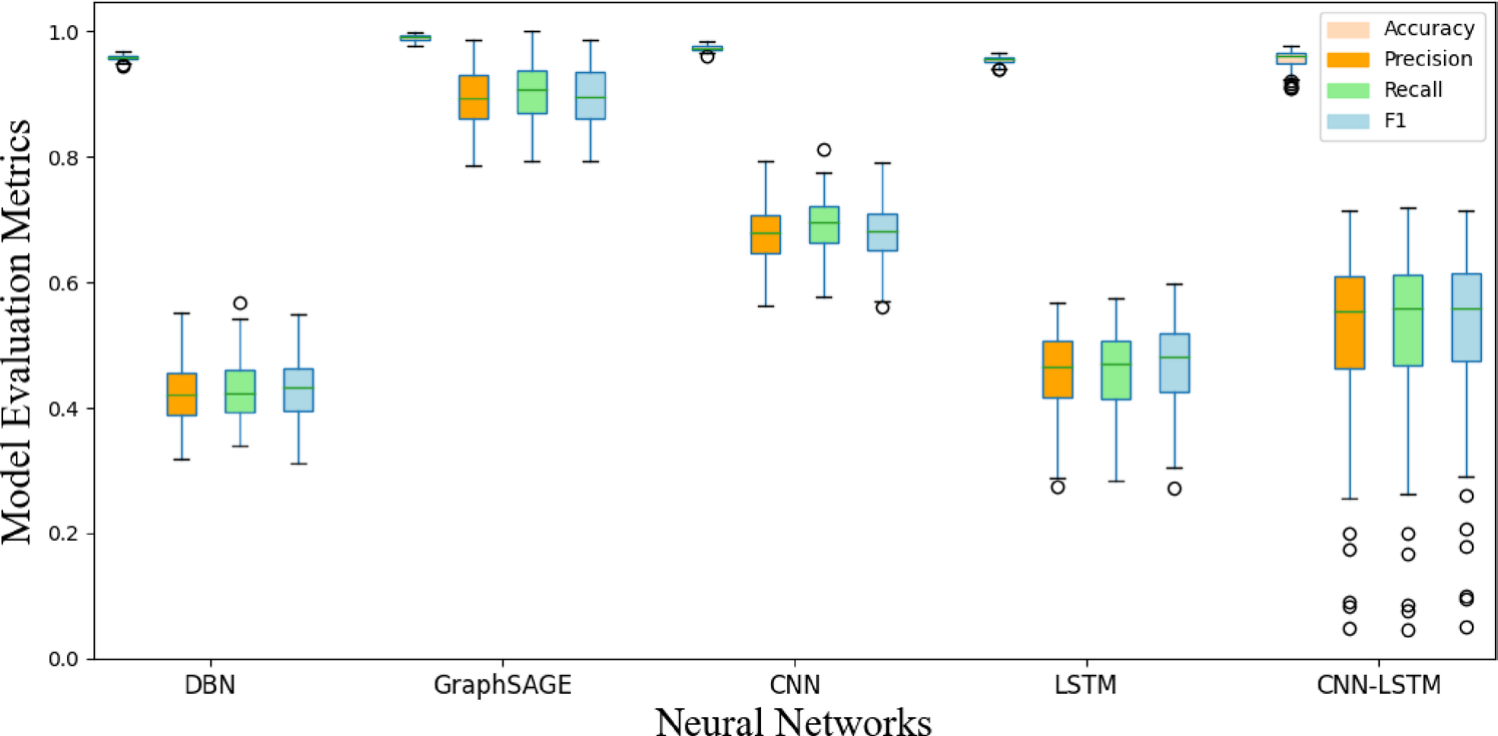
Performance comparison of different NN for locating abrupt faults.

**Fig. 15:**
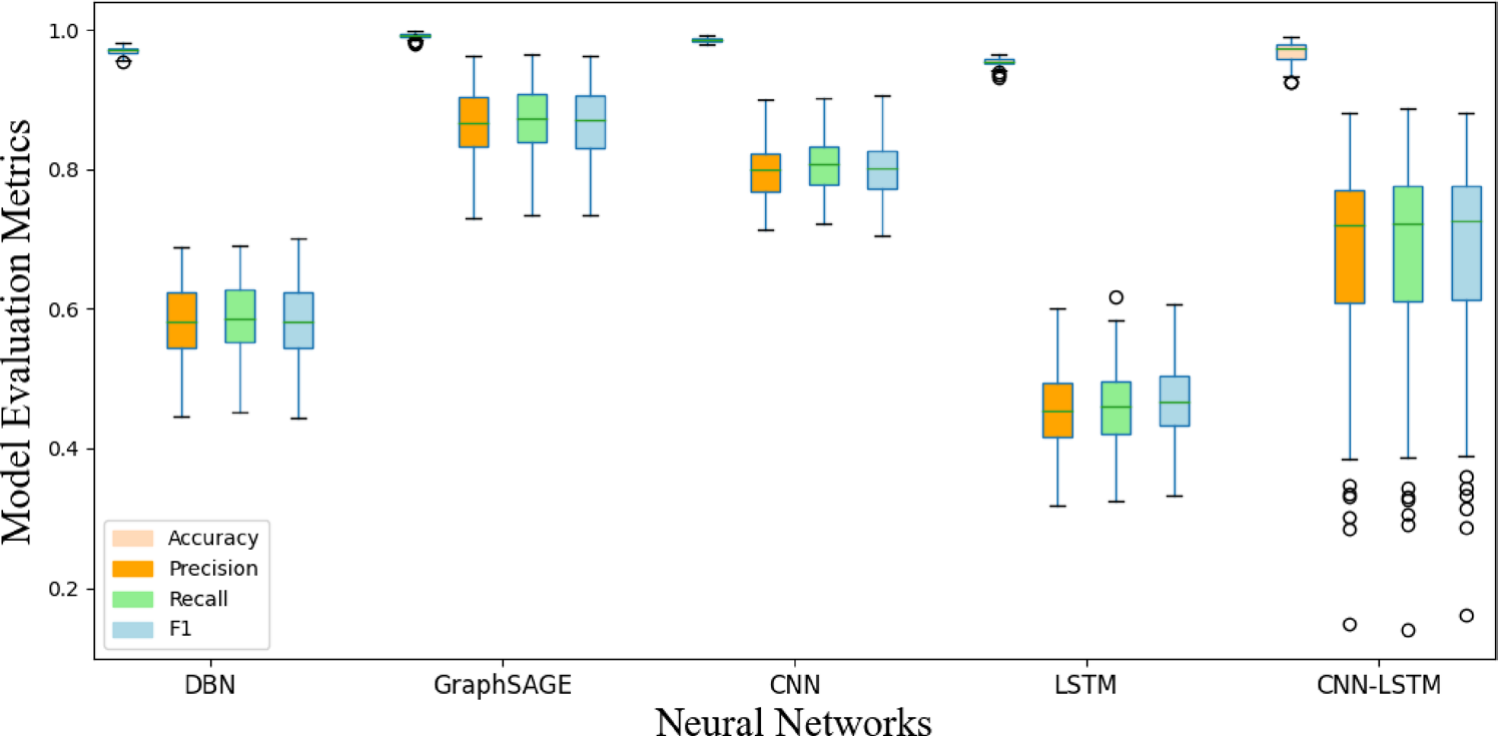
Performance comparison of different NN for locating slow faults.

Using the pack 1 dataset preprocessed with a window size of 3, we conducted a vertical comparison with other NN, and the results are presented in Figs. 14 and 15. Our model demonstrates optimal performance across all metrics, because the model establishes the connections between the batteries, reflecting the relational dependencies inherent in the battery system. It outperforms other models in both accuracy and generalization.

### Robustness verification

**Fig. 16:**
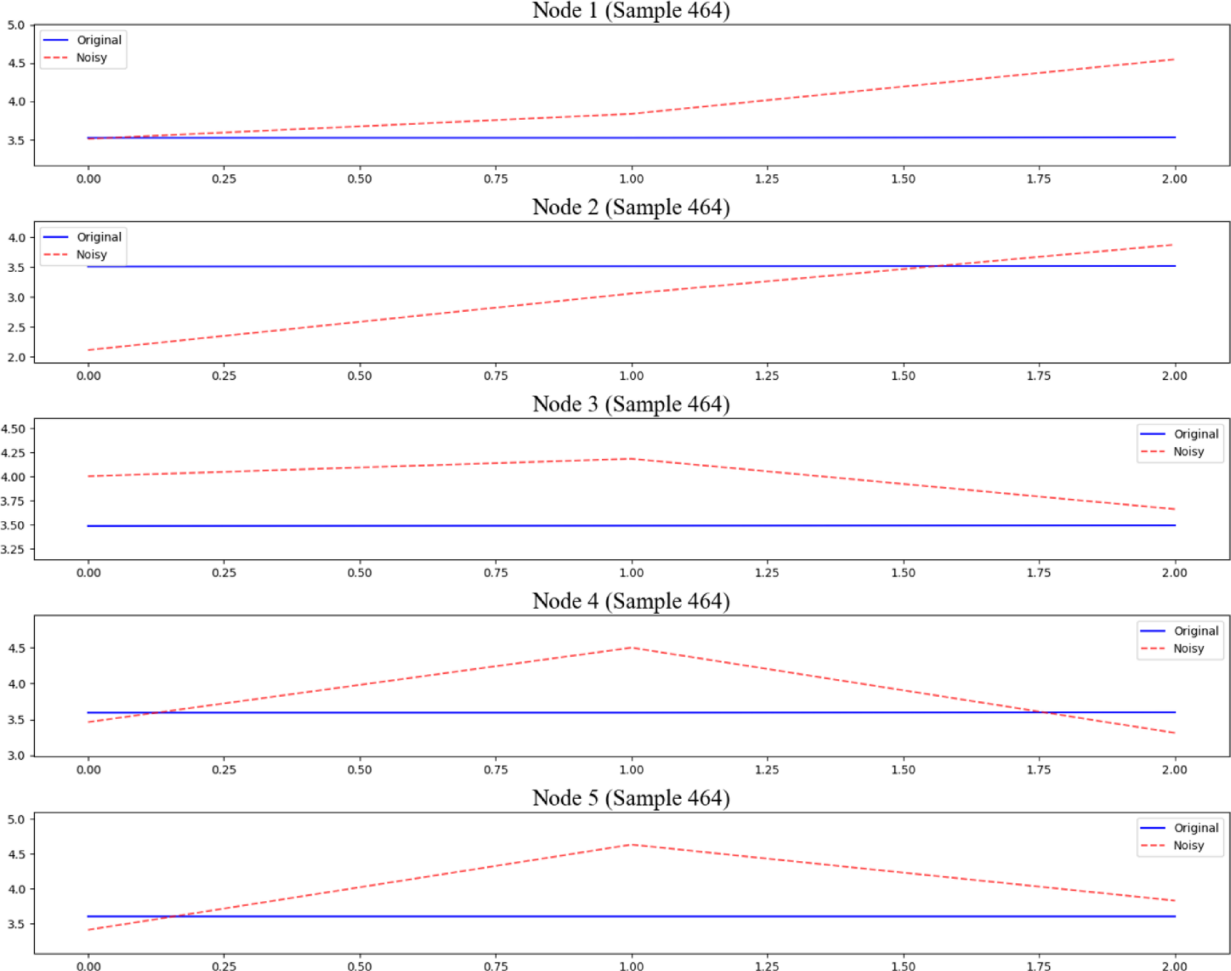
The comparison of disturbed data and normal data.

To validate the robustness of the proposed model under real-world perturbations, a systematic verification framework was designed, focusing on the model’s stability against Gaussian noise injection. The evaluation first simulated real-world interference scenarios by controlling noise addition. Specifically, Gaussian noise with a standard deviation of 0.5 (relative to the normalized data range) was randomly injected into 10% of the normal dataset. To isolate the impact of noise on the operational phase, fault samples were preserved while noise was introduced only into normal samples. This approach ensured that any performance degradation could be solely attributed to the injected perturbations, rather than inherent data biases. A comparison between disturbed data and normal data is shown in Fig. 16.

The disturbed dataset was then evaluated using the same model configuration as the original clean dataset. A strict comparison of performance metrics was conducted. The experimental results show that on the disturbed dataset, all metrics only slightly declined, indicating the model’s robustness. Specific metrics are shown in Table 5.

**Table 5:** Normal clean dataset compared to disturbed dataset.

Dataset type	Fault type	Accuracy	Precision	Recall	F1
Normal clean dataset	Abrupt fault	0.9990	0.9868	0.9868	0.9868
	Slow fault	0.9971	0.9644	0.9635	0.9619
Disturbed dataset	Abrupt fault	0.9980	0.9672	0.9672	0.9646
	Slow fault	0.9961	0.9426	0.9446	0.9403

## Conclusions

In this article, the proposed optimized GNN model extracts the relationships between various batteries by learning the topology of the batteries in the LIBs pack. The proposed method combines the physical coupling between batteries and the entanglement of measurement results with the strong nonlinear processing capability of NN to improve the effectiveness of fault localization. Compared to the highest-performing baseline method, the proposed method achieves the accuracy of abrupt fault and gradual localization. This indicates that the proposed optimized GNN method for diagnosing voltage faults has satisfactory accuracy and stability.

The fault diagnosis discussed in the article is only related to the voltage of the battery. Due to the complexity of LIBs systems, multiple faults often couple with each other and sometimes occur simultaneously. There is no universal fault diagnosis method that can be applied to all types of battery faults currently. Therefore, researchers are starting to diagnose different faults simultaneously, using simple rules to identify different faults with similar behavior. More research has also been conducted on the mechanism of battery failure, identifying more internal characteristics as fault indicators. Future work will focus on:

1) Developing diagnostic methods suitable for practical applications.

2) Multi-scale mechanism research between individual batteries, battery packs, and systems.

3) Research on multiple fault diagnosis methods.

4) Explore methods for considering multiple physical fields.

## Supplementary Information

Below is the link to the electronic supplementary material.

Supplementary Material 1

## Data Availability

1. Data generated or analysed during this study are included in supplementary information files. 2. The datasets used and/or analysed during the current study available from the corresponding author on reasonable request.
